# Incremental Prognostic Value of the Admission Systemic Immune-Inflammation Index Beyond BISAP for Early Risk Stratification of Acute Pancreatitis in the Emergency Department

**DOI:** 10.3390/jcm15176587

**Published:** 2026-08-26

**Authors:** Ömer Damar, Mahmut Yaman

**Affiliations:** 1 Department of Emergency Medicine, Mardin Artuklu University Faculty of Medicine, Mardin 47200, Türkiye; 2 Department of Emergency Medicine, Dicle University Faculty of Medicine, Diyarbakır 21280, Türkiye; drmahmutyaman@hotmail.com

**Keywords:** acute pancreatitis, systemic immune-inflammation index, BISAP score, risk stratification, prognosis, mortality

## Abstract

**Background:** Early identification of patients at risk of severe acute pancreatitis (AP) remains a clinical priority. We evaluated whether the admission Systemic Immune-Inflammation Index (SII, ×10^9^/L) adds prognostic value to the BISAP score, alone and in combination. SII integrates neutrophil, lymphocyte, and platelet counts from the routine complete blood count. **Methods:** In this retrospective single-center cohort, 523 adults with AP (January 2020–December 2025) were classified by the Revised Atlanta Classification. Admission SII and BISAP were related to severe AP, intensive care unit (ICU) admission, and in-hospital mortality using logistic regression, ROC analysis with DeLong comparison, 1000-sample bootstrap validation, and decision curve analysis. **Results:** Severe AP occurred in 48 patients (9.2%), ICU admission in 61 (11.7%), and death in 26 (5.0%). Admission SII was markedly higher in severe than non-severe disease (4085 vs. 1499; *p* < 0.001). Both SII and BISAP independently predicted all three outcomes after mutual adjustment (severe AP: SII odds ratio 2.12 and BISAP odds ratio 4.92 per standard deviation). For severe AP, the combined BISAP + SII model outperformed either marker (AUC 0.954 vs. 0.911 and 0.917), as it did for mortality (0.957); for ICU admission SII was the strongest single marker (0.869) and the combined model did not significantly improve discrimination compared with SII alone (DeLong *p* = 0.287). Internal validation showed adequate calibration with modest optimism and suggested potential net clinical benefit across the evaluated threshold ranges, pending external validation. **Conclusions:** Combining the admission SII with the BISAP score provides incremental prognostic information beyond BISAP alone for the early risk stratification of severe acute pancreatitis and in-hospital mortality using data already available at presentation. External, prospective validation is warranted before routine adoption.

## 1. Introduction

Acute pancreatitis (AP) is an acute inflammatory disorder of the pancreas and represents one of the leading causes of gastrointestinal-related hospital admissions worldwide. Over recent decades, its incidence has steadily increased, imposing a substantial clinical and economic burden on healthcare systems [1,2]. Although the majority of patients experience a mild, self-limiting disease course that resolves with supportive treatment, approximately 15–20% develop severe acute pancreatitis characterized by persistent organ failure, which is associated with markedly increased rates of intensive care unit (ICU) admission, local and systemic complications, and mortality [3,4]. Therefore, early identification of patients at high risk for disease progression is essential for timely fluid resuscitation, appropriate monitoring, optimal allocation of critical care resources, and early referral to tertiary care centers when necessary [3,5]. The 2012 Revised Atlanta Classification is currently the internationally accepted system for classifying disease severity and categorizes AP into mild, moderately severe, and severe forms based primarily on the presence and duration of organ failure [3]. This classification has provided a standardized framework for clinical practice and research; however, it was not designed as an early prognostic tool for risk stratification at hospital presentation. Consequently, there remains a need for simple, reliable, and readily available biomarkers that can accurately identify patients at increased risk of adverse clinical outcomes during the initial stage of the disease [3,6].

Early clinical risk stratification is a cornerstone of acute pancreatitis management, and several prognostic scoring systems have been developed to facilitate this process. Among these, the Bedside Index for Severity in Acute Pancreatitis (BISAP) score has gained widespread clinical acceptance because it consists of only five readily obtainable clinical variables, can be calculated within the first 24 h of admission, and does not require complex laboratory testing or advanced imaging [7]. Multiple systematic reviews and meta-analyses have demonstrated that the BISAP score provides good predictive performance for severe acute pancreatitis, organ failure, and mortality [7,8,9]. Nevertheless, accumulating evidence indicates that currently available clinical scoring systems alone do not achieve optimal discriminative ability for individualized risk prediction, highlighting the need for complementary biomarkers that reflect the underlying inflammatory response more comprehensively [6,8,10]. In parallel, increasing attention has been directed toward inflammation-based hematological indices that can be calculated from routine laboratory parameters. The Systemic Immune-Inflammation Index (SII), derived from platelet, neutrophil, and lymphocyte counts, has emerged as a novel biomarker reflecting the balance between host inflammatory activation and immune status. Previous studies have demonstrated its prognostic value across a wide range of clinical conditions, including malignancies, cardiovascular diseases, sepsis, and various inflammatory disorders [11,12,13]. More recently, several investigations have suggested that elevated SII values may also be associated with disease severity and adverse clinical outcomes in patients with acute pancreatitis [6,9]. However, most of this evidence has evaluated SII in isolation, and—despite BISAP’s well-documented ceiling on sensitivity—few studies have formally combined SII with BISAP within a single, fully specified multivariable model, reported the resulting model’s coefficients for independent application, or quantified its incremental discrimination, calibration, and net benefit against BISAP alone using contemporary prediction-model methodology. This gap, rather than the existence of SII as a prognostic marker per se, defines the specific contribution of the present study.

Therefore, the present study was designed to evaluate the prognostic performance of the admission Systemic Immune-Inflammation Index in comparison with, and in combination with, the BISAP score in patients presenting with acute pancreatitis. We hypothesized that admission SII would serve as an independent predictor of severe acute pancreatitis, ICU admission, and in-hospital mortality, and that integrating SII with the BISAP score would provide superior predictive performance compared with the BISAP score alone. Accordingly, the primary objective of this study was to assess the prognostic performance of SII and the BISAP score for predicting severe acute pancreatitis. The secondary objectives were to evaluate their prognostic value for ICU admission and in-hospital mortality and to compare the discrimination, calibration, and clinical utility of the combined BISAP + SII model. Accordingly, this study investigated whether combining an inflammation-based biomarker with an established clinical severity score could improve early risk stratification in patients with acute pancreatitis.

## 2. Materials and Methods

### 2.1. Study Population and Design

This retrospective observational cohort study was conducted in the emergency department of Mardin Training and Research Hospital, a tertiary-care hospital located in Mardin, Türkiye. The electronic medical records of consecutive adult patients diagnosed with acute pancreatitis between 1 January 2020, and 31 December 2025, were retrospectively reviewed. The study was designed and reported in accordance with the Strengthening the Reporting of Observational Studies in Epidemiology (STROBE) recommendations for observational cohort studies. Because this study also develops and internally validates a multivariable prediction model, it additionally followed the Transparent Reporting of a multivariable prediction model for Individual Prognosis or Diagnosis (TRIPOD) statement [14]; the completed TRIPOD checklist is provided as a separate Supporting File. Consistent with TRIPOD terminology, the combined BISAP + SII model reported here is a prediction-model development study with internal (bootstrap) validation; it is not, at this stage, externally validated, and is not intended or recommended for clinical implementation.

Acute pancreatitis was diagnosed when at least two of the following three criteria were present: (1) characteristic acute upper abdominal pain; (2) serum amylase or lipase activity at least three times the upper limit of the normal reference range; and (3) imaging findings consistent with acute pancreatitis on ultrasonography, contrast-enhanced computed tomography, or magnetic resonance imaging. The diagnosis was established at the time of initial emergency department presentation using the criteria above, not retrospectively at the time of discharge, and was subsequently confirmed during retrospective chart review using the available clinical, biochemical, and radiological data.

Because the study was retrospective and involved no modification of routine patient management, no study-specific intervention was performed. All diagnostic investigations, treatment decisions, intensive care unit admissions, and discharge decisions were made by the treating physicians according to the institutional clinical practice in effect during the study period.

### 2.2. Participant Selection

All consecutive patients aged 18 years or older who presented during the study period and fulfilled the diagnostic criteria for acute pancreatitis were screened for eligibility. Consecutive sampling was used to reduce selection bias and to reflect the clinical spectrum of acute pancreatitis encountered in routine emergency care.

Patients were included when they were aged 18 years or older, fulfilled at least two accepted diagnostic criteria for acute pancreatitis, presented to or were evaluated at the study center within 24 h of symptom onset, had admission complete blood count data permitting calculation of the Systemic Immune-Inflammation Index, had sufficient clinical and laboratory data to calculate the BISAP score, and had sufficient follow-up information to determine disease severity, ICU admission, and in-hospital survival status.

Patients were excluded if they were younger than 18 years, had chronic or recurrent pancreatitis, were transferred to the study center more than 24 h after symptom onset, or had incomplete clinical or laboratory data required for the primary analyses. Patients with missing values for SII, BISAP, Revised Atlanta severity classification, ICU admission, or in-hospital mortality were not included in the final analytic cohort.

Patients with a diagnosis of COVID-19 were not included in the study cohort at any stage of patient identification: during the study period, patients with confirmed SARS-CoV-2 infection were managed through a separate institutional pathway and were not captured within the standard emergency-department acute-pancreatitis registry from which the present cohort was drawn, so that none of the 701 screened patients had an active COVID-19 diagnosis.

A total of 701 consecutive patients were screened. Of these, 178 were excluded because of age younger than 18 years (*n* = 22), chronic or recurrent pancreatitis (*n* = 71), transfer more than 24 h after symptom onset (*n* = 36), or incomplete clinical data (*n* = 49). The final cohort therefore comprised 523 patients. These 523 patients represented 523 unique individuals, each identified by a single, unique hospital protocol number; no repeat admissions were identified in or included in the analytic cohort.

### 2.3. Data Collection and Quality Control

Demographic, clinical, and laboratory data were retrospectively extracted from the electronic medical records by a board-certified emergency physician using a standardized data collection form. To ensure data accuracy and consistency, all extracted data were independently reviewed and verified by a second board-certified emergency physician. Any discrepancies were resolved by re-examining the original medical records until consensus was reached. The extracted variables included age, sex, time from symptom onset to admission, etiology of pancreatitis, comorbidities, admission vital signs, Glasgow Coma Scale score, complete blood count parameters, biochemical test results, imaging findings (including posteroanterior chest radiography and contrast-enhanced CT imaging findings), BISAP score components, ICU admission, length of hospital stay, and in-hospital mortality.

For patients with repeated measurements, only the first values obtained at the initial hospital presentation were used to calculate the admission SII and BISAP score. Laboratory values obtained later during hospitalization were not used for the primary prognostic analyses. Data were entered into an electronic database and checked for range errors, implausible values, duplicate records, and internal inconsistencies. Any discrepancies identified during data verification were resolved by re-examination of the original electronic medical records.

No statistical imputation was performed for variables required to calculate the main predictors or define the study outcomes. Patients lacking the data necessary to calculate SII or BISAP or to determine the primary or secondary outcomes were excluded from the complete-case analysis.

### 2.4. Laboratory Measurements

Venous blood samples were collected as part of routine clinical assessment at the time of initial hospital presentation. Per standing departmental triage protocol, blood is drawn at presentation before intravenous fluid resuscitation or other pancreatitis-directed therapeutic intervention is initiated; this sequence was followed for the large majority of patients in this cohort, so that BISAP components and the complete blood count underlying SII predominantly reflect pre-treatment values, although individual-patient timestamps confirming this sequence were not independently verified retrospectively. Complete blood count measurements, including white blood cell, neutrophil, lymphocyte, and platelet counts and hemoglobin concentration, were performed using an automated hematology analyzer (Sysmex XN series, Sysmex Corporation, Kobe, Japan) in the hospital’s central laboratory.

Serum glucose, blood urea nitrogen, creatinine, albumin, C-reactive protein, amylase, lipase, lactate, and triglyceride concentrations were measured using an automated biochemistry analyzer (Cobas 8000 modular analyzer, Roche Diagnostics, Mannheim, Germany) with the manufacturer’s validated reagents. Procalcitonin concentrations were determined using a commercially available automated immunoassay system (Cobas e 601 module, Roche Diagnostics, Mannheim, Germany) according to the manufacturer’s instructions. The same analytical platforms and reference ranges were used throughout the study period, with routine internal and external quality-control procedures applied continuously to maintain analytical consistency across the six-year study period.

Laboratory personnel were blinded to the objectives of the present retrospective study because all measurements were obtained as part of routine clinical care. Reference intervals and analytical measurement ranges were those routinely used by the institutional central laboratory during the study period.

### 2.5. Calculation of SII and BISAP Score

The Systemic Immune-Inflammation Index, expressed in units of ×10^9^/L, was calculated from the admission complete blood count using the following formula:

$$SII=\frac{\mathrm{platelet}\;\mathrm{count}\times \mathrm{neutrophil}\;\mathrm{count}}{\mathrm{lymphocyte}\;\mathrm{count}}$$

Platelet, neutrophil, and lymphocyte counts obtained from the same admission blood sample were used in the calculation. SII (expressed in units of ×10^9^/L, equivalently ×10^3^/μL, based on cell counts in the same units) was analyzed as a continuous variable in the primary regression and receiver operating characteristic analyses. For logistic regression modeling, continuous SII values were standardized as z scores so that the reported odds ratios represented the change in outcome odds associated with each one-standard-deviation increase.

The BISAP score was calculated using five variables, each determined from the first values available at initial hospital presentation, consistent with the ascertainment of SII: blood urea nitrogen greater than 25 mg/dL, impaired mental status defined as a Glasgow Coma Scale score below 15, the presence of systemic inflammatory response syndrome (defined per the 1992 ACCP/SCCM consensus criteria as at least two of: temperature above 38 °C or below 36 °C; heart rate above 90/min; respiratory rate above 20/min or PaCO_2_ below 32 mmHg; and white blood cell count above 12,000/mm^3^, below 4000/mm^3^, or more than 10% immature bands), age greater than 60 years, and the presence of pleural effusion, assessed by postero-anterior chest radiography obtained at the time of initial emergency department presentation and distinct from the later contrast-enhanced computed tomography used for pancreatic morphology and necrosis characterization. One point was assigned for each component, producing a total score ranging from 0 to 5. The same scoring definition was applied to all patients.

### 2.6. Disease Severity and Outcome Definitions

Disease severity was classified according to the 2012 Revised Atlanta Classification. Mild acute pancreatitis was defined by the absence of organ failure and local or systemic complications. Moderately severe acute pancreatitis was defined by transient organ failure resolving within 48 h and/or the presence of local or systemic complications without persistent organ failure. Severe acute pancreatitis was defined by persistent organ failure lasting longer than 48 h. Organ failure was assessed using the modified Marshall scoring system and was considered present when a score of at least 2 was recorded for the respiratory, cardiovascular, or renal system.

For the principal binary analysis, patients with mild and moderately severe acute pancreatitis were combined into the non-severe group, whereas patients fulfilling the Revised Atlanta criteria for severe acute pancreatitis constituted the severe group. This definition corresponds to the grouping used in the disease-severity analyses presented in the manuscript.

The primary outcome was severe acute pancreatitis according to the Revised Atlanta Classification.

The secondary outcomes were admission to the intensive care unit during the index hospitalization and all-cause in-hospital mortality before discharge.

ICU admission was determined from hospital admission and transfer records. In-hospital mortality was defined as death from any cause occurring during the index hospitalization. Patients discharged alive were classified as survivors. The outcomes were determined from the complete hospitalization record without post-discharge follow-up.

### 2.7. Sample Size

Because this was a retrospective cohort study, no patient recruitment was undertaken specifically for research purposes. All consecutive patients meeting the prespecified eligibility criteria during the study period were included, and no formal a priori sample-size calculation was used to restrict the cohort.

The final sample included 523 patients, among whom 48 developed severe acute pancreatitis, 61 required ICU admission, and 26 died during hospitalization. The multivariable models were deliberately restricted to two prespecified predictors, SII and BISAP, to limit model overfitting, particularly for the mortality outcome with the smallest number of events. Internal validation with bootstrap resampling was additionally performed to quantify optimism in model discrimination and calibration.

### 2.8. Statistical Analysis

Statistical analyses were performed using IBM SPSS Statistics for Windows, version 26.0 (IBM Corp., Armonk, NY, USA) and R software version 4.5.3 (R Foundation for Statistical Computing, Vienna, Austria). The distribution of continuous variables was assessed using the Shapiro–Wilk test together with visual inspection of histograms and quantile–quantile plots. Normally distributed variables are presented as mean ± standard deviation (SD) and were compared using the independent-samples *t* test, whereas non-normally distributed variables are expressed as median (interquartile range [IQR]) and were compared using the Mann–Whitney U test. Categorical variables are presented as number (percentage) and were compared using the Pearson χ^2^ test or Fisher’s exact test, as appropriate.

Univariable binary logistic regression analyses were initially performed for SII and BISAP separately. Prespecified multivariable logistic regression models were subsequently constructed by simultaneously entering SII and BISAP for each study outcome. Continuous predictors were standardized before model fitting; therefore, odds ratios (ORs) are reported per one-standard-deviation increment with corresponding 95% confidence intervals (CIs). Model performance was evaluated using the likelihood-ratio χ^2^ test, Nagelkerke’s R^2^, the Akaike information criterion (AIC), and the Hosmer–Lemeshow goodness-of-fit test.

Model discrimination was assessed using receiver operating characteristic (ROC) curve analysis, and areas under the curve (AUCs) with 95% confidence intervals were calculated. Pairwise comparisons between ROC curves were performed using the DeLong method. For the combined BISAP + SII model, each outcome was modelled by multivariable logistic regression in which the BISAP score entered linearly and SII was fitted with a three-knot restricted cubic spline to accommodate a potentially non-linear association; the model’s predicted probabilities were then used as the combined score for ROC, calibration, and decision-curve analyses. The spline-based combined model was used throughout for ROC, calibration, and decision-curve analyses, whereas the mutually adjusted linear model entered both BISAP and SII as linear (standardized) terms to yield interpretable per-standard-deviation odds ratios; the two models serve different purposes and their outputs should not be conflated. Spline knots were placed at the 10th, 50th, and 90th percentiles of the observed SII distribution (771, 1600, and 3907 ×10^9^/L, respectively), yielding 2 degrees of freedom for SII (1 linear plus 1 spline term) in addition to 1 degree of freedom for BISAP; the nonlinear spline term was statistically significant for all three outcomes (*p* = 0.004–0.014), supporting a nonlinear SII–outcome relationship. Full model coefficients, including the spline coding and the equation used to calculate predicted probabilities, are reported in Supplementary Table S1. Optimal cutoff values for individual predictors were determined using the maximum Youden index, and sensitivity, specificity, positive predictive value (PPV), negative predictive value (NPV), positive likelihood ratio (LR+), and negative likelihood ratio (LR−) were calculated.

Calibration of the combined models was evaluated primarily using the Brier score, calibration-in-the-large intercept, calibration slope, and graphical calibration plots; the Hosmer–Lemeshow test was reported as a supplementary, not primary, goodness-of-fit check, given its recognized instability with sparse outcomes. Graphical calibration plots were constructed by grouping patients into 10 equal-sized bins of predicted probability and plotting the mean predicted versus mean observed outcome per bin, without additional smoothing. Internal validation was performed using 1000 bootstrap resamples according to the Harrell approach to estimate optimism-corrected model performance: in each iteration, patients were resampled with replacement, SII spline knots were recalculated from the resample’s own 10th/50th/90th percentiles, the full BISAP + SII model was refit on the resample, and performance was evaluated both on the resample (apparent) and on the original cohort (test); optimism was defined as the difference between these two estimates, and the optimism-corrected estimate was the original apparent value minus the mean optimism across the 1000 iterations. Because the apparent calibration intercept is evaluated in the same sample used for model development, a value at or near zero is an expected property of maximum-likelihood logistic regression and does not, by itself, constitute evidence of external calibration. Decision curve analysis (DCA) was conducted to evaluate the net clinical benefit of SII, BISAP, and the combined model across a range of clinically relevant threshold probabilities. For clinical interpretation of the decision curves, attention was focused on threshold probabilities of 5–30% for severe acute pancreatitis and ICU admission and 1–20% for in-hospital mortality; these exploratory ranges were selected to encompass clinically plausible escalation thresholds around the observed event frequencies for each outcome and should not be interpreted as externally validated decision thresholds. All statistical tests were two-sided, and a *p* value <0.05 was considered statistically significant. ROC curve comparisons, bootstrap validation, calibration analyses, and decision curve analyses were performed in R using the pROC, rms, and rmda packages.

## 3. Results

**Figure 1:**
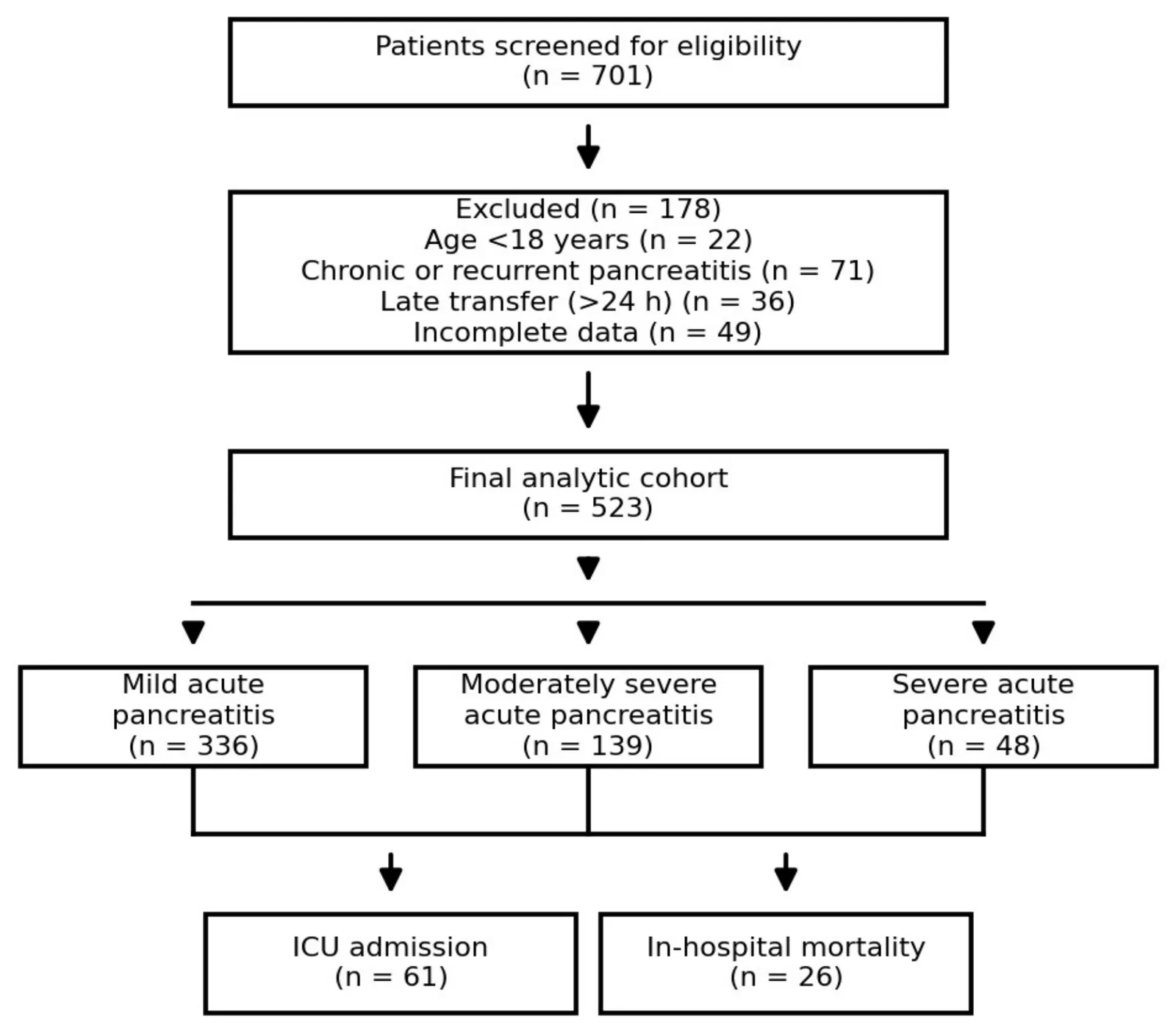
Study flow diagram (STROBE). Patient selection, eligibility, and classification by Revised Atlanta severity.

A total of 701 consecutive patients with acute pancreatitis were screened for eligibility. After exclusion of 178 patients because of age <18 years (*n* = 22), chronic or recurrent pancreatitis (*n* = 71), transfer more than 24 h after symptom onset (*n* = 36), or incomplete clinical data (*n* = 49), 523 patients were included in the final analysis. According to the Revised Atlanta classification, 336 patients (64.2%) had mild acute pancreatitis, 139 (26.6%) had moderately severe disease, and 48 (9.2%) developed severe acute pancreatitis. During hospitalization, 61 patients (11.7%) required intensive care unit (ICU) admission, whereas 26 (5.0%) died before discharge (Figure 1).

The mean age of the study population was 59.5 ± 16.3 years, and 267 patients (51.1%) were male. Biliary pancreatitis was the most frequent etiology (60.2%), followed by idiopathic (15.5%), other causes (8.8%), alcohol-related disease (7.8%), and hypertriglyceridemia (7.6%). The median time from symptom onset to hospital admission was 17.4 h (IQR, 11.7–21.7). The median admission SII was 1548 (IQR, 1057–2300), whereas the median BISAP score was 2.0 (IQR, 1.0–3.0). Additional baseline demographic, clinical, laboratory, and outcome characteristics are summarized in Table 1.

Patients with severe acute pancreatitis were significantly older than those with non-severe disease (69.8 ± 15.9 vs. 58.5 ± 16.0 years, *p* < 0.001). Diabetes mellitus and chronic kidney disease were more prevalent among patients with severe disease, whereas the distribution of etiologic factors did not differ significantly between groups.

At presentation, severe acute pancreatitis was characterized by more pronounced physiological derangement, including lower systolic blood pressure and oxygen saturation together with higher heart rate and respiratory rate. Patients with severe disease also had lower Glasgow Coma Scale scores than those with non-severe pancreatitis (all *p* < 0.001).

Laboratory evaluation demonstrated substantially greater systemic inflammatory activation in severe disease. Compared with non-severe pancreatitis, severe cases exhibited significantly higher white blood cell and neutrophil counts, lower lymphocyte counts, higher glucose, blood urea nitrogen, creatinine, C-reactive protein, procalcitonin, and lactate concentrations, together with lower albumin and hemoglobin levels (all *p* ≤ 0.006). Platelet count did not differ significantly between groups (*p* = 0.485). Admission SII (×10^9^/L) was markedly higher in patients with severe acute pancreatitis than in those with non-severe disease (4085 vs. 1499, *p* < 0.001). Likewise, BISAP scores were significantly higher in severe disease (median 4.0 vs. 2.0, *p* < 0.001) (Table 2).

Both admission SII and BISAP scores increased consistently across all adverse clinical outcomes. Patients who required ICU admission had substantially higher SII values than those managed outside the ICU (3765 vs. 1481, *p* < 0.001), accompanied by higher BISAP scores (3.0 vs. 2.0, *p* < 0.001). Similarly, patients who died during hospitalization demonstrated markedly elevated SII values (3776 vs. 1535, *p* < 0.001) and higher BISAP scores (4.0 vs. 2.0, *p* < 0.001). Parallel differences were observed for severe acute pancreatitis, consistently linking a higher admission inflammatory burden to subsequent adverse outcomes (Table 3).

In univariable analyses, both admission SII and BISAP score were significant predictors of severe acute pancreatitis, ICU admission, and in-hospital mortality (all *p* < 0.001). After simultaneous adjustment for each other in multivariable logistic regression models, both variables remained independently associated with all three study outcomes.

For prediction of severe acute pancreatitis, BISAP score demonstrated the strongest independent association (OR 4.92, 95% CI 3.04–7.96), whereas SII also remained independently predictive (OR 2.12, 95% CI 1.57–2.86). Similar independent associations were observed for ICU admission (BISAP: OR 2.25, 95% CI 1.59–3.17; SII: OR 2.29, 95% CI 1.73–3.04) and in-hospital mortality (BISAP: OR 5.75, 95% CI 3.08–10.71; SII: OR 1.55, 95% CI 1.13–2.14). The Hosmer–Lemeshow tests showed no evidence of gross lack of fit for the mutually adjusted linear models (all *p* > 0.05), with Nagelkerke R^2^ values ranging from 0.387 to 0.535 (Table 4).

Receiver operating characteristic analysis demonstrated excellent discrimination of both SII and BISAP for severe acute pancreatitis, ICU admission, and in-hospital mortality. For severe acute pancreatitis, SII achieved an AUC of 0.911, whereas BISAP yielded an AUC of 0.917. Combining both variables significantly improved discrimination, resulting in an AUC of 0.954.

**Figure 2:**
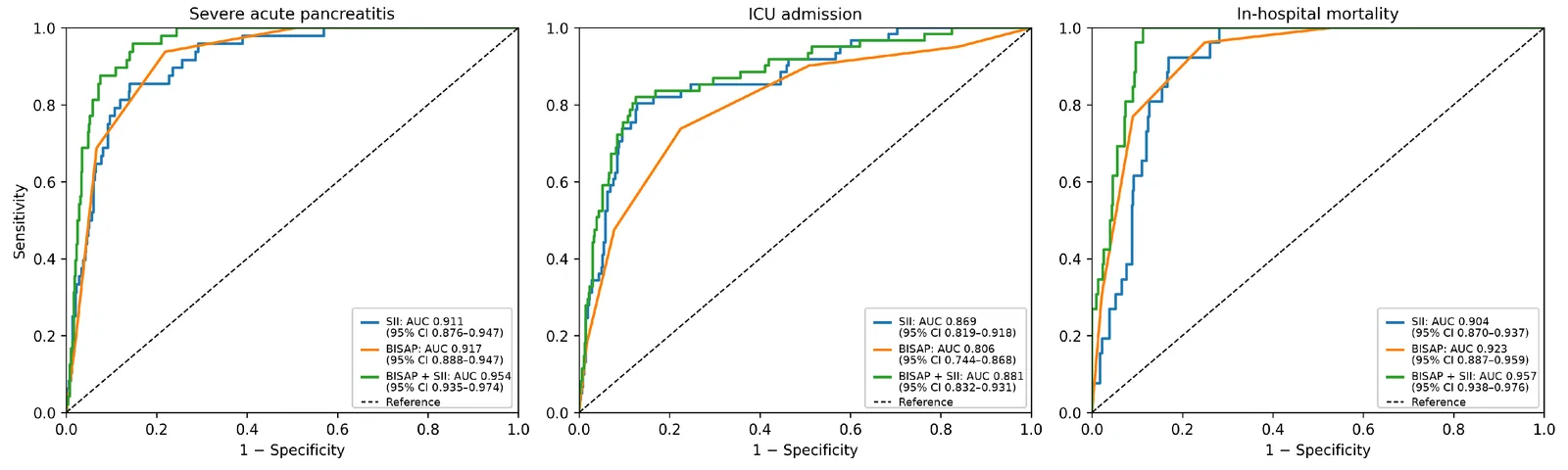
Receiver operating characteristic curves of the SII, BISAP score, and the combined BISAP + SII model for prediction of severe acute pancreatitis, ICU admission, and in-hospital mortality.

For ICU admission, SII alone demonstrated the highest performance among individual markers (AUC 0.869), whereas the combined model achieved a comparable AUC of 0.881. DeLong testing indicated that the combined model significantly outperformed BISAP alone but not SII alone (*p* = 0.287). In contrast, for in-hospital mortality, integration of SII with BISAP increased discrimination from AUCs of 0.904 and 0.923 for the individual markers to 0.957 for the combined model. DeLong analyses confirmed that the combined model significantly exceeded the performance of either marker alone for both severe acute pancreatitis and mortality (all *p* < 0.05) (Figure 2 and Table 5 and Table 6).

**Figure 3:**
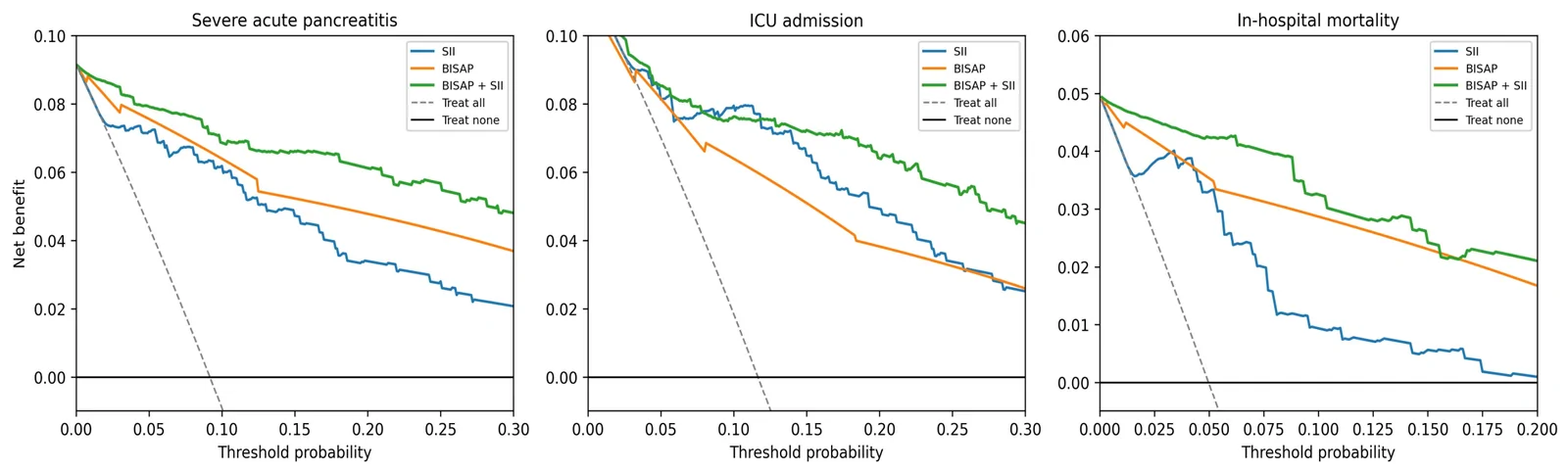
Decision curve analysis for (left to right) severe acute pancreatitis, ICU admission, and in-hospital mortality, comparing the SII, BISAP score, and the combined BISAP + SII model against the treat-all and treat-none strategies. Net benefit is plotted across clinically relevant threshold probabilities: 5–30% for severe acute pancreatitis and ICU admission (intensified monitoring, critical-care consultation, or ICU-bed request) and 1–20% for in-hospital mortality (enhanced monitoring, senior-clinician review, or higher-acuity assessment); axes are capped and scaled accordingly for each outcome.

**Figure 4:**
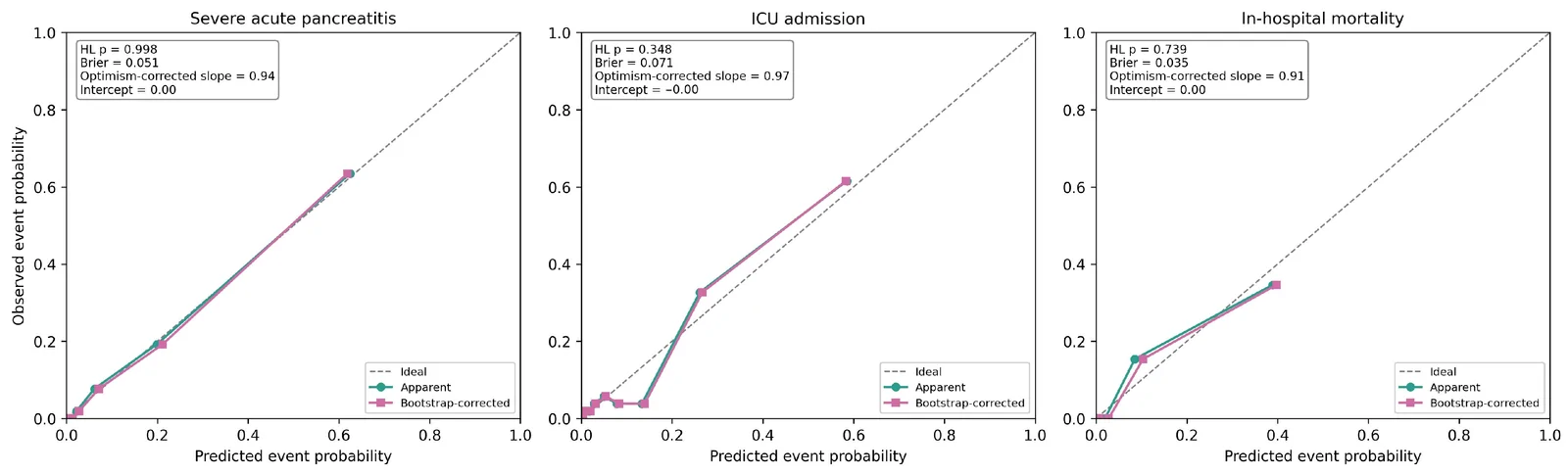
Calibration plots of the BISAP + SII model for (left to right) severe acute pancreatitis, ICU admission, and in-hospital mortality. The plots show the apparent model performance and the bootstrap-corrected calibration (1000 bootstrap resamples; Harrell method) against the ideal calibration line.

Internal validation demonstrated adequate calibration with modest optimism on bootstrap resampling. Optimism-corrected calibration slopes were 0.94 (severe acute pancreatitis), 0.97 (ICU admission), and 0.91 (in-hospital mortality); calibration intercepts were approximately zero, and bootstrap-corrected AUCs differed only modestly from the apparent AUCs (Table 6), indicating limited optimism overall, with somewhat greater optimism for the mortality model consistent with its lower events-per-variable ratio (see Limitations). Decision curve analysis suggested that the combined BISAP + SII model generally provided greater net clinical benefit across the evaluated threshold ranges, particularly for severe acute pancreatitis and in-hospital mortality. For ICU admission, the combined model and SII alone showed broadly comparable net benefit over part of the evaluated threshold range, consistent with the ROC findings (Figure 3). Calibration plots confirmed close agreement between predicted and observed risks for all three outcomes (Figure 4).

## 4. Discussion

In this retrospective cohort of 523 patients with acute pancreatitis, the admission Systemic Immune-Inflammation Index (SII) and the BISAP score were each independently associated with severe disease, intensive care unit (ICU) admission, and in-hospital mortality. More importantly, combining the two into a single BISAP + SII model raised discrimination for severe acute pancreatitis from an AUC of 0.917 for BISAP alone to 0.954, and for mortality from 0.923 to 0.957, with the added benefit confirmed by DeLong testing. The combined model showed adequate internal calibration and, in decision-curve analysis, suggested greater net benefit than either predictor alone across the evaluated threshold ranges; these findings require external validation. Taken together, these findings suggest that a routinely available hematological index and an established bedside score are not competing tools but complementary ones.

The independent predictive weight we observed for the BISAP score is consistent with a large body of evidence. Pooled meta-analytic data have repeatedly placed the discriminative accuracy of BISAP for severe acute pancreatitis in the 0.87–0.94 range [6,8,9], and single-center cohorts have reported values as high as 0.96 for severity prediction [15]. Our apparent AUC of 0.917 for BISAP therefore sits comfortably within the reported spectrum rather than at its optimistic edge. What the literature has also made clear, however, is the score’s characteristic profile: high specificity paired with only modest sensitivity. A meta-analysis of ten studies found that a BISAP threshold of ≥3 predicted severe disease with a specificity around 91% but a sensitivity near 51% [8], meaning that a substantial minority of patients who ultimately deteriorate are not flagged at the cut-off. This is precisely the gap that a continuously distributed inflammatory marker is well positioned to fill.

SII behaved in our cohort exactly as its biological rationale would predict. Because it integrates neutrophil, lymphocyte, and platelet counts into a single ratio, it captures two processes that unfold in parallel during severe pancreatic injury: neutrophil-driven amplification of the systemic inflammatory response and the relative lymphopenia that accompanies stress and immune exhaustion [10,11]. Admission SII was more than doubled in patients with severe disease compared with non-severe disease (4085 vs. 1499), and rose in step across every adverse outcome we examined. This dose–response pattern echoes earlier work. Liu and colleagues first proposed SII as an early indicator of severity in acute pancreatitis [16], and a subsequent MIMIC-based analysis linked higher admission SII to increased 30- and 90-day mortality, with the highest tertile carrying more than a twofold hazard [17]. Additional retrospective cohorts, including emergency-department populations, have likewise linked a higher admission SII to greater AP severity and worse clinical outcomes, affirming SII as a reproducible prognostic signal rather than a single-center curiosity [18,19,20].

SII was chosen over the neutrophil-to-lymphocyte ratio (NLR) because it additionally incorporates the platelet count, capturing the thrombo-inflammatory axis alongside the neutrophil-lymphocyte balance; platelets contribute to local microvascular thrombosis and systemic inflammatory amplification implicated in acute pancreatitis severity, even though platelet counts alone did not differ significantly between groups in univariable comparison (Table 2). Head-to-head comparisons of SII versus NLR specific to acute pancreatitis remain limited, and we identify this as a useful direction for future study; NLR values were available in our dataset and could support such a comparison in subsequent work. As a further exploratory analysis, we examined whether C-reactive protein and procalcitonin, two established inflammatory markers in pancreatitis, added independent information beyond the BISAP + SII model: when entered simultaneously into the combined model, procalcitonin remained an independent predictor for all three outcomes (severe acute pancreatitis *p* < 0.001, ICU admission *p* = 0.012, mortality *p* = 0.020), whereas the independent contribution of C-reactive protein was weaker and not statistically significant for severe acute pancreatitis (*p* = 0.76) once BISAP, SII, and procalcitonin were accounted for. We did not incorporate procalcitonin into the primary composite score because our design goal was a model restricted to components of BISAP and a routine complete blood count—both universally and rapidly available in virtually any emergency department—whereas procalcitonin has more limited availability and longer turnaround time in many settings; combining procalcitonin with SII + BISAP is a promising direction for future model refinement and prospective validation.

The most clinically meaningful message of our analysis lies not in either marker alone but in their combination. For severe acute pancreatitis and for in-hospital mortality, the BISAP + SII model significantly outperformed both individual predictors, whereas for ICU admission SII alone was already the strongest single marker and the combined model offered no statistically significant gain over it (DeLong *p* = 0.287). We read this divergence as informative rather than inconsistent. ICU admission is a decision partly driven by resource availability, institutional admission policies, and clinician judgment, and it appears to track the inflammatory burden that SII quantifies more closely than the physiological derangement captured by BISAP. Severity and death, by contrast, are biological endpoints shaped by both dimensions, and it is here that a marker of inflammation and a marker of organ dysfunction reinforce each other. The idea that a composite of an inflammatory index and a clinical score can exceed either component has precedent: combined CRP–NLR scoring has been shown to triple the odds of correctly identifying organ failure in acute pancreatitis [21], and simple hematological ratios paired with severity scores have repeatedly approached or matched more cumbersome systems such as Ranson or APACHE II [7,22,23].

These findings suggest that the complementary strengths of inflammatory biomarkers and clinical severity scores may provide a more comprehensive assessment of early disease progression than either approach alone. Several practical implications follow. SII requires nothing beyond a complete blood count that is already drawn in essentially every patient presenting to an emergency department, and it can be computed within minutes of admission—well before the 24 h window that BISAP itself requires for full ascertainment [7]—without additional laboratory resources or delay to clinical decision-making. In a district or resource-limited setting, where APACHE II is impractical and advanced imaging may be delayed, a bedside score augmented by a readily available laboratory index offers a pragmatic route to earlier risk stratification [10]. This matters because the global incidence of acute pancreatitis has risen steadily over recent decades, and although overall mortality is near 1%, it climbs to 30–40% once organ failure or necrosis supervenes—the very subgroup early stratification aims to capture [2,4]. Our decision curve analysis extends this argument from discrimination to net benefit: DCA suggested that, under the evaluated threshold assumptions, the combined model could provide greater net benefit than either predictor alone across a clinically relevant range of threshold probabilities. Calibration slopes near unity and modest optimism on internal bootstrap validation provide some reassurance against overfitting, although this should be confirmed by external validation before the finding is considered established.

It is worth being explicit about what our data do and do not support. Both SII and BISAP performed strongly, but neither replaces clinical vigilance, and the incremental value of SII was outcome-dependent rather than universal. We also caution against extrapolating these results to populations we did not study—patients with chronic or recurrent pancreatitis, those presenting beyond 24 h, and children were all excluded by design, and the reported cut-offs should not be transported uncritically to cohorts with a different case mix or etiologic distribution. Notably, the prognostic behavior of hematological indices can vary by etiology, and NLR-type markers have been shown to perform differently in hypertriglyceridemia-induced versus biliary disease [24,25]; our predominantly biliary cohort should be kept in mind when generalizing.

Several methodological refinements should be considered in future work. Serial SII measurements over the first 48–72 h, rather than a single admission value, may capture prognostically relevant trajectories of inflammation, consistent with longitudinal data on NLR [22]. Prospective recording of established benchmark scores (Ranson, APACHE II) and a validated CRP timepoint would allow the combined model to be positioned against the broader field of severity scores rather than against BISAP alone. Multicenter prospective validation across etiologic subgroups is needed to confirm the robustness and generalizability of the reported cut-offs.

## 5. Limitations

First, the single-center, retrospective design drawing on a cohort in which biliary disease predominated constrains the external validity of the specific SII cut-offs we report, since the discriminative behavior of inflammation-based indices is known to shift with etiologic composition.

Second, only 48 patients met the criteria for severe disease and 26 died, so the multivariable models—although deliberately restricted to two prespecified predictors and internally validated by bootstrap resampling to limit overfitting—rest on a modest number of events. This constraint is most pronounced for the mortality model, whose events-per-variable ratio of 8.7 falls below the conventional threshold of 10 often recommended for stable logistic regression; bootstrap internal validation nonetheless demonstrated only modest optimism for this outcome (apparent AUC 0.957, optimism-corrected AUC 0.951), but external validation in a larger mortality cohort is particularly warranted.

Third, we captured a single admission SII value rather than serial measurements and did not record contemporaneous Ranson or APACHE II scores, which prevented us from characterizing the trajectory of inflammation over time or benchmarking the combined model against the full range of established severity systems, and external validation was not available; the proposed model should therefore be confirmed in independent multicenter cohorts before widespread clinical implementation.

Fourth, by definition these 49 patients were missing one or more of the variables required for the primary analyses—components of the admission complete blood count needed to calculate SII, BISAP component data, Revised Atlanta severity classification, or ascertainment of ICU admission or in-hospital mortality—but patient-level detail on which specific variable(s) were missing for each patient, and a direct comparison of baseline characteristics between included and excluded patients, could not be performed because this information was not retained in the final research database. Because we lack patient-level information on why each of these 49 records was incomplete, the direction and magnitude of any resulting selection bias cannot be reliably estimated from our data; plausible mechanisms in a retrospective single-center cohort could act in either direction (e.g., very brief encounters or early transfer tending to exclude milder patients, versus early death before complete data capture tending to exclude the most unstable patients), but we did not verify which, if any, applied here, and we report this as an unresolved limitation rather than an estimated bias.

Fifth, patients with active COVID-19 infection were not represented in this cohort, as they were managed through a separate clinical pathway during the study period; we were therefore unable to evaluate the potential interaction between SARS-CoV-2 infection and the systemic immune-inflammation index in the context of acute pancreatitis, which represents an important direction for future investigation in cohorts that include such patients.

## 6. Conclusions

In this retrospective cohort of patients with acute pancreatitis, both the admission Systemic Immune-Inflammation Index and the BISAP score were independent predictors of severe disease, intensive care unit admission, and in-hospital mortality. Integrating the two into a single BISAP + SII model improved discrimination for severe acute pancreatitis and for in-hospital mortality beyond either measure alone; for intensive care unit admission, SII performed strongly on its own and the combination added no further discrimination. The combined model showed promising internally validated calibration and net benefit across clinically relevant thresholds, but its clinical utility and transportability require prospective external validation before this can be considered established. Because SII is derived from the complete blood count already obtained on virtually all patients at presentation, combining it with BISAP is, in principle, feasible without requiring additional testing or turnaround time; however, this combined model was developed in a single retrospective cohort without external validation, and its incremental value should be considered exploratory until confirmed prospectively before any recommendation for routine clinical implementation. The combined BISAP + SII model may have potential to support early risk stratification using routinely available clinical and laboratory information. These findings apply to the population studied here and warrant external, ideally prospective and multicenter, validation before the reported thresholds are adopted in routine practice.

## Figures and Tables

**Table 1:** Baseline characteristics of the study population (*N* = 523).

Characteristic	Total (*N* = 523)
**Demographics**	
Age, years	59.5 ± 16.3
Time from symptom onset to admission, h	17.4 [11.7–21.7]
Male sex	267 (51.1)
**Etiology**	
Biliary	315 (60.2)
Alcohol	41 (7.8)
Hypertriglyceridemia	40 (7.6)
Idiopathic	81 (15.5)
Other	46 (8.8)
**Comorbidities**	
Hypertension	159 (30.4)
Diabetes mellitus	165 (31.5)
Chronic kidney disease	35 (6.7)
**Admission vital signs**	
Systolic blood pressure, mmHg	121.0 [109.0–135.0]
Heart rate, beats/min	92.8 ± 16.6
Respiratory rate, breaths/min	19.0 [16.0–22.5]
Oxygen saturation, %	95.0 [93.0–97.0]
Glasgow Coma Scale	15.0 [14.0–15.0]
**Admission laboratory findings**	
White blood cell count, ×10^3^/µL	12.7 [10.1–15.7]
Neutrophil count, ×10^3^/µL	9.2 [7.4–12.0]
Lymphocyte count, ×10^3^/µL	1.5 ± 0.5
Platelet count, ×10^3^/µL	258.3 ± 64.2
Hemoglobin, g/dL	13.2 ± 1.8
Glucose, mg/dL	147.0 [115.0–174.0]
Blood urea nitrogen, mg/dL	21.6 [15.4–29.3]
Creatinine, mg/dL	1.1 [0.7–1.5]
Albumin, g/dL	3.6 [3.4–4.0]
C-reactive protein, mg/L	45.2 [29.8–80.0]
Procalcitonin, ng/mL	0.2 [0.1–0.6]
Amylase, U/L	648 [517–919]
Lipase, U/L	1362 [1122–1926]
Lactate, mmol/L	1.8 [1.1–2.5]
Triglyceride, mg/dL	184.0 [121.0–308.0]
**Clinical score**	
Systemic immune-inflammation index, ×10^9^/L	1548 [1057–2300]
BISAP score	2.0 [1.0–3.0]
Modified Marshall score	1.0 [0.0–2.0]
**Clinical outcomes**	
Severe acute pancreatitis	48 (9.2)
ICU admission	61 (11.7)
In-hospital mortality	26 (5.0)
Length of hospital stay, days	6.0 [3.0–10.0]

Data are presented as mean ± standard deviation, median [interquartile range], or *n* (%). Normality was assessed with the Shapiro–Wilk test. BISAP, Bedside Index for Severity in Acute Pancreatitis; ICU, intensive care unit; SII, systemic immune-inflammation index (platelet × neutrophil/lymphocyte).

**Table 2:** Comparison of baseline characteristics according to disease severity (Revised Atlanta classification).

Characteristic	Non-Severe (*n* = 475)	Severe (*n* = 48)	*p* Value
**Demographics**			
Age, years	58.5 ± 16.0	69.8 ± 15.9	<0.001
Time from symptom onset to admission, h	17.2 [11.6–21.7]	19.4 [12.8–21.9]	0.638
Male sex	242 (50.9)	25 (52.1)	1.000
**Etiology (overall *p* = 0.749)**			
Biliary	284 (59.8)	31 (64.6)	0.623
Alcohol	38 (8.0)	3 (6.2)	1.000
Hypertriglyceridemia	38 (8.0)	2 (4.2)	0.566
Idiopathic	72 (15.2)	9 (18.8)	0.655
Other	43 (9.1)	3 (6.2)	0.788
**Comorbidities**			
Hypertension	140 (29.5)	19 (39.6)	0.198
Diabetes mellitus	141 (29.7)	24 (50.0)	0.006
Chronic kidney disease	26 (5.5)	9 (18.8)	0.001
**Admission vital signs**			
Systolic blood pressure, mmHg	123.0 [110.0–137.0]	110.5 [100.8–120.2]	<0.001
Heart rate, beats/min	90.8 ± 15.6	112.3 ± 13.2	<0.001
Respiratory rate, breaths/min	19.0 [15.0–22.0]	26.0 [23.0–28.2]	<0.001
Oxygen saturation, %	95.0 [93.0–97.0]	91.0 [90.0–93.0]	<0.001
Glasgow Coma Scale	15.0 [14.0–15.0]	14.0 [13.0–14.0]	<0.001
**Admission laboratory findings**			
White blood cell count, ×10^3^/µL	12.3 [10.0–14.8]	18.4 [16.2–20.3]	<0.001
Neutrophil count, ×10^3^/µL	9.0 [7.3–11.3]	14.7 [12.9–16.6]	<0.001
Lymphocyte count, ×10^3^/µL	1.5 ± 0.5	0.9 ± 0.3	<0.001
Platelet count, ×10^3^/µL	257.7 ± 63.9	264.9 ± 67.9	0.485
Hemoglobin, g/dL	13.3 ± 1.8	12.5 ± 1.8	0.006
Glucose, mg/dL	142.0 [111.5–168.0]	198.0 [172.8–217.8]	<0.001
Blood urea nitrogen, mg/dL	20.6 [14.9–27.0]	32.8 [26.8–40.1]	<0.001
Creatinine, mg/dL	1.0 [0.7–1.4]	2.0 [1.5–2.2]	<0.001
Albumin, g/dL	3.7 [3.4–4.0]	3.0 [2.6–3.3]	<0.001
C-reactive protein, mg/L	42.8 [28.4–70.0]	98.0 [81.9–132.2]	<0.001
Procalcitonin, ng/mL	0.2 [0.1–0.5]	1.8 [1.1–3.0]	<0.001
Lactate, mmol/L	1.6 [1.0–2.2]	4.0 [3.6–4.6]	<0.001
**Clinical score**			
Systemic immune-inflammation index (SII) ×10^9^/L	1499 [1033–2231]	4085 [3101–6052]	<0.001
BISAP score	2.0 [1.0–2.0]	4.0 [3.0–4.0]	<0.001
Modified Marshall score	1 [0–2]	4 [2–5]	**<0.001**

Non-severe acute pancreatitis comprises the mild and moderately severe Revised Atlanta categories; severe denotes the severe category. Continuous variables were compared with the independent-samples *t*-test or Mann–Whitney U test; categorical variables (*n* (%)) with the χ^2^ or Fisher exact test. The *p* value for etiology represents the overall comparison across all etiologic categories. Abbreviations as in Table 1.

**Table 3:** Admission SII and BISAP score according to each study outcome.

Outcome	Event	No Event	*p*
**Severe acute pancreatitis**	**Severe (*n* = 48)**	**Non-severe (*n* = 475)**	
SII	4085 [3101–6052]	1499 [1033–2231]	<0.001
BISAP score	4.0 [3.0–4.0]	2.0 [1.0–2.0]	<0.001
**ICU admission**	**ICU (*n* = 61)**	**No ICU (*n* = 462)**	
SII	3765 [2809–5525]	1481 [1014–2199]	<0.001
BISAP score	3.0 [2.0–4.0]	2.0 [1.0–2.0]	<0.001
**In-hospital mortality**	**Died (*n* = 26)**	**Survived (*n* = 497)**	
SII	3776 [3094–5336]	1535 [1058–2290]	<0.001
BISAP score	4.0 [4.0–5.0]	2.0 [1.0–2.0]	<0.001

Data are presented as median [interquartile range] and compared using the Mann–Whitney U test. BISAP, Bedside Index for Severity in Acute Pancreatitis; ICU, intensive care unit; SII, systemic immune-inflammation index.

**Table 4:** Logistic regression analysis of mutually adjusted linear SII and BISAP predictors.

Predictor	Univariable OR (95% CI)	*p*	Multivariable OR (95% CI) *	*p*
**Severe acute pancreatitis (events, *n* = 48)**				
BISAP score	7.05 (4.45–11.19)	<0.001	4.92 (3.04–7.96)	<0.001
SII, ×10^9^/L	3.22 (2.43–4.26)	<0.001	2.12 (1.57–2.86)	<0.001
*Model fit: likelihood-ratio χ^2^(2) = 147.0, p < 0.001; Nagelkerke R^2^ = 0.535; Hosmer–Lemeshow p = 0.825; AIC = 179.7*				
**ICU admission (events, *n* = 61)**				
BISAP score	3.44 (2.52–4.69)	<0.001	2.25 (1.59–3.17)	<0.001
SII, ×10^9^/L	3.11 (2.38–4.08)	<0.001	2.29 (1.73–3.04)	<0.001
*Model fit: likelihood-ratio χ^2^(2) = 115.7, p < 0.001; Nagelkerke R^2^ = 0.387; Hosmer–Lemeshow p = 0.263; AIC = 267.0*				
**In-hospital mortality (events, *n* = 26)**				
BISAP score	7.29 (4.05–13.11)	<0.001	5.75 (3.08–10.71)	<0.001
SII, ×10^9^/L	2.36 (1.80–3.09)	<0.001	1.55 (1.13–2.14)	0.007
*Model fit: likelihood-ratio χ^2^(2) = 82.1, p < 0.001; Nagelkerke R^2^ = 0.445; Hosmer–Lemeshow p = 0.838; AIC = 130.7*				

Odds ratios are expressed per 1-standard-deviation increment after z-standardization of each continuous predictor. Continuous predictors were standardized before model fitting. * Each multivariable model includes the BISAP score and SII together. Model-fit statistics refer to the multivariable model for each outcome. CI, confidence interval; OR, odds ratio; AIC, Akaike information criterion. Other abbreviations as in Table 1. This linear model is distinct from the restricted-cubic-spline combined model used for Table 5 and Table 6 and described in Section 2.8.

**Table 5:** Receiver operating characteristic analysis of the SII, BISAP score, and their combination.

Predictor	AUC (95% CI)	Cut-Off (95% CI)	Sens. (95% CI)	Spec. (95% CI)	PPV (95% CI)	NPV (95% CI)	LR+	LR−	Youden
**Severe acute pancreatitis**									
SII	0.911 (0.876–0.947)	2629.4 (2066.6–3117.4)	0.85 (0.73–0.93)	0.86 (0.82–0.89)	0.38 (0.29–0.47)	0.98 (0.97–0.99)	6.06	0.17	0.71
BISAP	0.917 (0.888–0.947)	≥3	0.94 (0.83–0.98)	0.78 (0.74–0.82)	0.30 (0.23–0.38)	0.99 (0.98–1.00)	4.28	0.08	0.72
BISAP + SII	0.954 (0.935–0.974)	0.087	0.96 (0.86–0.99)	0.85 (0.82–0.88)	0.40 (0.31–0.49)	1.00 (0.98–1.00)	6.50	0.05	0.81
**ICU admission**									
SII	0.869 (0.819–0.918)	2629.4 (2432.3–2975.3)	0.80 (0.69–0.88)	0.87 (0.84–0.90)	0.45 (0.36–0.55)	0.97 (0.95–0.98)	6.29	0.23	0.68
BISAP	0.806 (0.744–0.868)	≥3	0.74 (0.62–0.83)	0.77 (0.73–0.81)	0.30 (0.23–0.38)	0.96 (0.93–0.97)	3.28	0.34	0.51
BISAP + SII	0.881 (0.832–0.931)	0.174	0.82 (0.71–0.90)	0.87 (0.84–0.90)	0.46 (0.37–0.56)	0.97 (0.95–0.99)	6.53	0.21	0.69
**In-hospital mortality**									
SII	0.904 (0.870–0.937)	2629.4 (2214.8–3072.0)	0.92 (0.76–0.98)	0.83 (0.80–0.86)	0.22 (0.15–0.31)	1.00 (0.98–1.00)	5.46	0.09	0.75
BISAP	0.923 (0.887–0.959)	≥3	0.96 (0.81–0.99)	0.75 (0.71–0.79)	0.17 (0.12–0.24)	1.00 (0.99–1.00)	3.85	0.05	0.71
BISAP + SII	0.957 (0.938–0.976)	0.062	1.00 (0.87–1.00)	0.89 (0.86–0.91)	0.32 (0.23–0.42)	1.00 (0.99–1.00)	8.88	0.00	0.89

Optimal cut-offs were derived from the Youden index; for the combined model the cut-off refers to the predicted probability. Because the SII has a single distribution and its discriminative ability concentrated around a similar threshold for all severe outcomes, the independent Youden analyses yielded the same optimal SII cut-off (2629.4) for severe acute pancreatitis, ICU admission, and in-hospital mortality. Bootstrap 95% confidence intervals for the SII cut-off (shown in the Cut-off column for SII) confirm this stability across 1000 resamples; this threshold should be regarded as exploratory, pending external validation, rather than a validated decision threshold. The 95% confidence intervals for the AUC were computed with the DeLong method, and 95% confidence intervals for sensitivity, specificity, PPV, and NPV were computed using the Wilson method. For ICU admission, SII was the strongest single marker and the combined BISAP + SII model did not improve on SII alone (DeLong *p* = 0.287); for severe acute pancreatitis and mortality the combined model was superior to both markers alone (DeLong *p* < 0.05 for each; see Table 6). AUC, area under the curve; CI, confidence interval (Wilson method for Sens., Spec., PPV, NPV; DeLong method for AUC; percentile bootstrap for the SII cut-off); LR+, positive likelihood ratio; LR−, negative likelihood ratio; NPV, negative predictive value; PPV, positive predictive value; Sens., sensitivity; Spec., specificity. Other abbreviations as in Table 1.

**Table 6:** Performance and internal validation of the BISAP + SII model for each outcome.

Outcome	AUC	*p* vs. BISAP *	*p* vs. SII *	ΔAUC vs. BISAP-Only (95% CI)	LR Test vs. BISAP-Only (χ^2^, *p*)	HL *p*	Brier	Slope **	Intercept	Boot. AUC **
Severe acute pancreatitis	0.954	<0.001	0.001	0.035 (0.012–0.059)	χ^2^ = 41.43, *p* <0.0001	0.998	0.051	0.94	0.00	0.950
ICU admission	0.881	<0.001	0.287	0.075 (0.037–0.114)	χ^2^ = 50.00, *p* <0.0001	0.348	0.071	0.97	−0.00	0.879
In-hospital mortality	0.957	0.005	0.002	0.034 (0.009–0.059)	χ^2^ = 23.88, *p* <0.0001	0.739	0.035	0.91	0.00	0.951

* DeLong tests compare the BISAP + SII model against BISAP alone and against SII alone. For severe acute pancreatitis and mortality, the combined model was superior to both single markers; for ICU admission it improved on BISAP but not on SII (*p* = 0.287). ΔAUC and the likelihood-ratio (LR) test directly compare the BISAP + SII model against a BISAP-only model (nested-model comparison, 2 degrees of freedom for all three outcomes); across all three outcomes, addition of SII produced a statistically significant improvement in discrimination by both criteria. The Hosmer–Lemeshow test is known to be unstable when expected cell counts are sparse, as for in-hospital mortality (26 events over 10 risk deciles); calibration was therefore also assessed graphically, which corroborated adequate calibration for all three outcomes. ** Optimism-corrected AUC and calibration slope from 1000-sample bootstrap internal validation (Harrell method); the calibration intercept (calibration-in-the-large) is reported on the apparent predictions. A lower Brier score, a calibration slope near 1.0, and an intercept near 0 indicate better calibration; the HL *p* value is reported as a supplementary goodness-of-fit statistic. Abbreviations are shown in Table 1 and Table 5.

## Data Availability

The aggregate data presented in this study are available within the article. The patient-level dataset analyzed in this study is not publicly available because of institutional data-sharing restrictions, but de-identified data and analysis code may be made available by the corresponding author upon reasonable request and institutional approval.

## Supplementary Materials

The following supporting information can be downloaded at: https://www.mdpi.com/article/10.3390/jcm15176587/s1, Table S1: Regression coefficients of the BISAP + SII combined model (restricted cubic spline for SII) for each outcome; File S1: TRIPOD Checklist.
