# Supplementary material for: Incremental Prognostic Value of the Admission Systemic Immune-Inflammation Index Beyond BISAP for Early Risk Stratification of Acute Pancreatitis in the Emergency Department
Source: J Clin Med. 2026 Aug 26;15(17):6587. doi: 10.3390/jcm15176587 (PMC13566213; doi:10.3390/jcm15176587)
Supplement: Supplementary file 1 [file jcm-15-06587-s001.zip › Supplementary Materials.pdf]

Supplementary Materials

Manuscript Title: *Incremental Prognostic Value of the Admission Systemic Immune-Inflammation Index Beyond BISAP for Early Risk Stratification of Acute Pancreatitis in the Emergency Department*
Journal: Journal of Clinical Medicine

Table S1. Regression coefficients of the BISAP + SII combined model (restricted cubic spline for SII) for each outcome.

Outcome	β0 (Intercept)	β1 (BISAP)	β2 (SII, linear)	β3 (SII, spline)
Severe acute pancreatitis	−13.879244 ($p < 0.001$)	1.098236 ($p < 0.001$)	0.004498 ($p = 0.002$)	−0.005488 ($p = 0.004$)
ICU admission	−7.956201 ($p < 0.001$)	0.545680 ($p < 0.001$)	0.002465 ($p < 0.001$)	−0.002765 ($p = 0.004$)
In-hospital mortality	−23.624862 ($p = 0.001$)	1.116897 ($p < 0.001$)	0.009022 ($p = 0.013$)	−0.011526 ($p = 0.014$)

Predicted probability: $\text{logit}(P) = \beta0 + \beta1\times\text{BISAP} + \beta2\times\text{SII} + \beta3\times\text{SII\_spline}$, with $P = 1/(1+e^{-\text{logit}(P)})$. SII_spline is the single nonlinear basis term of a 3-knot restricted cubic spline (Harrell coding) fitted to the raw SII value, with knots at the 10th, 50th, and 90th percentiles of the observed SII distribution (771, 1,600, and 3,907 $\times10^9$/L, respectively). Explicitly, with knots t1=771, t2=1600, t3=3907 (all $\times10^9$/L) and (u)+ denoting the positive part of u (i.e., u if u>0, else 0), $\text{SII\_spline} = [(\text{SII}-t1)_+^3 - (\text{SII}-t2)_+^3\times(t3-t1)/(t3-t2) + (\text{SII}-t3)_+^3\times(t2-t1)/(t3-t2)] / (t3-t1)^2$, following the standard Harrell restricted-cubic-spline parameterization for a single interior term with 3 knots. ΔAUC and likelihood-ratio statistics comparing this model with a BISAP-only model are reported in Table 6 of the main manuscript.
