# Supplementary material for: Incremental Prognostic Value of the Admission Systemic Immune-Inflammation Index Beyond BISAP for Early Risk Stratification of Acute Pancreatitis in the Emergency Department
Source: J Clin Med. 2026 Aug 26;15(17):6587. doi: 10.3390/jcm15176587 (PMC13566213; doi:10.3390/jcm15176587)
Supplement: Supplementary file 1 [file jcm-15-06587-s001.zip › TRIPOD Checklist.pdf]

TRIPOD Checklist: Prediction Model Development
Manuscript ID: jcm-4478893
Title: *Incremental Prognostic Value of the Admission Systemic Immune-Inflammation Index Beyond BISAP for Early Risk Stratification of Acute Pancreatitis in the Emergency Department*
TRIPOD type: 1a — model development only, with internal (bootstrap) validation; no external validation cohort.

Section	Item	Checklist Item	Reported on Page	Location / Notes
Title	1	Identify the study as developing a multivariable prediction model, the target population, and the outcome to be predicted.	1	Partially reported: the title identifies the population, predictors (SII, BISAP), and outcomes, but does not explicitly use the terms “prediction model,” “development,” or “internal validation”; this framing is made explicit instead in the Abstract and Section 2.1 (p. 3).
Abstract	2	Provide a summary of objectives, design, setting, participants, sample size, predictors, outcome, statistical analysis, results, and conclusions.	1	Structured Abstract (Background/Methods/Results/Conclusions).
Introduction	3a	Explain the medical context and rationale for developing the prediction model.	1–2	Introduction, paragraphs 1–2.
Introduction	3b	Specify the objectives, including whether the study describes model development, validation, or both.	2–3	Introduction, final paragraph; explicitly framed as development with internal validation (Section 2.1, p. 3).
Methods	4a	Describe the study design and date of data collection.	3	Section 2.1: retrospective cohort, Jan 2020–Dec 2025.
Methods	4b	Specify key study dates.	3	Section 2.1.
Methods	5a	Describe the setting, eligible participants, and data sources.	3–4	Sections 2.1–2.2: single ED, Mardin Training and Research Hospital.
Methods	5b	Describe eligibility criteria.	3	Section 2.2, inclusion/exclusion criteria.
Methods	5c	Give details of treatment received, if relevant.	3	Section 2.1: no study-specific intervention; routine care.
Methods	6a	Clearly define the outcome predicted and how/when assessed.	3, 6	Sections 2.1 and 2.6: severe AP (Revised Atlanta), ICU admission, in-hospital mortality.
Methods	6b	Report blinding of outcome assessment.	4	No prospective blinding of outcome assessment to predictor values was performed or possible, as this was a retrospective single-source chart review in which predictor and outcome data resided in the same record; instead, all extracted data (predictors and outcomes together) were independently re-abstracted and cross-checked by a second physician, with discrepancies resolved against the source record (Section 2.3), which we report as a data-accuracy safeguard rather than a blinding procedure.
Methods	7a	Clearly define all predictors used.	4–5	Sections 2.3–2.5: SII and BISAP components.
Methods	7b	Report blinding of predictor assessment.	5	Section 2.4: laboratory personnel who generated the complete blood count and biochemistry results were blinded to the study's objectives (i.e., unaware their assays

				would be used for this analysis), which minimizes measurement-related bias; this is distinct from, and does not substitute for, blinding of predictor assessment to outcome status, which was not performed in this single-record retrospective design.
Methods	8	Explain how the study size was arrived at.	6	Section 2.7 (Sample Size): consecutive-sampling rationale; no formal a priori calculation.
Methods	9	Describe how missing data were handled.	4, 19	Section 2.3 (complete-case analysis); Limitations Fourth (49 excluded patients, missingness discussion).
Methods	10a	Describe how predictors were handled in the analyses.	6	Section 2.8: continuous predictors, standardization for Table 4; combined model specification.
Methods	10b	Specify type of model, all model-building procedures, and method for internal validation.	6–7	Section 2.8: logistic regression, restricted cubic spline, 1000-sample bootstrap (Harrell method); clinical rationale for DCA threshold ranges.
Methods	10d	Specify all measures used to assess model performance and, if relevant, to compare multiple models.	6–7, 12–16	Section 2.8 specifies AUC/DeLong, calibration (Brier, intercept, slope, HL), and decision-curve analysis as the performance measures; results reported in Tables 5–6 and Figures 2–4.
Risk groups	11	Provide details on how risk groups were created, if done.	13	Section 3/Table 5: Youden-optimal cut-offs, presented as exploratory.
Methods	12	For validation, identify differences from the development data (N/A — development-only study).	—	Not applicable; no external validation cohort (declared in Section 2.1 and Limitations Third, p. 18).
Results	13a	Describe the flow of participants, with reasons for exclusion.	10	Section 3, Figure 1 (STROBE flow diagram).
Results	13b	Describe characteristics of participants.	8–10	Section 3, Tables 1 and 2.
Results	14a	Report the number of participants and outcome events.	7–8	Section 3, para. 1: 523 patients; 48/61/26 events.
Results	14b	If done, report unadjusted association between predictors and outcome.	11	Table 3, Table 4 (univariable columns).
Results	15a	Present the full prediction model to allow predictions for individuals.	Separate Supplementary Materials file	Supplementary Table S1 (separate document, per MDPI back-matter format): intercept, coefficients, spline coding, explicit spline basis formula, predicted-probability equation.
Results	15b	Explain how to use the model.	6; Supplementary Materials file	Section 2.8 (model specification) and Supplementary Table S1 (equation, in the separate Supplementary Materials document).
Results	16	Report model performance measures (discrimination, calibration).	12–16	Tables 4–6, Figures 2 and 4.
Results	18	Discuss limitations, including sources of potential bias and imprecision.	18–19	Section 5, Limitations (5 items).
Discussion	19b	Interpret results with reference to objectives, prior evidence, and specifically as a development (not yet validated) study.	16–18	Section 4, Discussion (development-model framing, Section 2.1, carried through).
Discussion	20	Discuss the potential clinical use and implications for future research, avoiding overinterpretation.	16–19	Limitations and Conclusions (exploratory framing; external validation called for).

Other	21	Provide information on availability of supplementary resources.	19	Supplementary Materials statement (p. 19) and separate Supplementary Materials / TRIPOD checklist files.
Other	22	Give the source of funding and role of funders.	19	Funding statement: no specific funding received.

Reference: Collins GS, Reitsma JB, Altman DG, Moons KG. Transparent reporting of a multivariable prediction model for individual prognosis or diagnosis (TRIPOD): the TRIPOD statement. BMJ. 2015;350:g7594.
